# The Concurrent Detection of Chelonid Alphaherpesvirus 5 and *Chelonia mydas* Papillomavirus 1 in Tumoured and Non-Tumoured Green Turtles

**DOI:** 10.3390/ani11030697

**Published:** 2021-03-05

**Authors:** Narges Mashkour, Karina Jones, Wytamma Wirth, Graham Burgess, Ellen Ariel

**Affiliations:** 1 College of Public Health, Medical and Veterinary Sciences, James Cook University, Townsville, QLD 4814, Australia; karina.jones@jcu.edu.au (K.J.); wytamma.wirth@my.jcu.edu.au (W.W.); graham.burgess@jcu.edu.au (G.B.); ellen.ariel@jcu.edu.au (E.A.); 2 College of Medicine and Dentistry, James Cook University, Townsville, QLD 4814, Australia

**Keywords:** marine turtles, tumour, fibropapillomatosis, Chelonid alphaherpesvirus 5, *Chelonia mydas* papillomavirus 1

## Abstract

**Simple Summary:**

Characterised by benign tumours, fibropapillomatosis is a debilitating disease that predominantly afflicts the endangered green turtle (*Chelonia mydas*). A growing body of evidence has associated these tumours with a herpesvirus. However, a recent study detected both herpesvirus and papillomavirus in these tumours. This result challenged the idea that the herpesvirus is the sole virus associated with this disease. The present study aimed to better understand the co-occurrence of these viruses in turtles with fibropapillomatosis (in both tumour samples and non-tumoured skin samples), in addition to samples from non-tumoured turtles. Both viruses were detected in all sample types, with the 43.5% of tumours containing both herpesvirus and papillomavirus. Tumour samples were found to contain the most herpesvirus while the highest amount of papillomavirus was detected in non-tumoured skin from turtles with tumours. Collectively, these results pivot the way we think about this disease; as an infectious disease where two separate viruses may be at play.

**Abstract:**

Characterised by benign tumours, fibropapillomatosis (FP) is a debilitating disease that predominantly afflicts the endangered green turtle (*Chelonia mydas*). A growing body of histological and molecular evidence has associated FP tumours with Chelonid alphaherpesvirus 5 (ChHV5). However, a recent study which detected both ChHV5 and *Chelonia mydas* papillomavirus 1 (CmPV1) DNA in FP tumour tissues has challenged this hypothesis. The present study aimed to establish a probe-based qPCR to assess the wider prevalence of CmPV1 and co-occurrence with ChHV5 in 275 marine turtles foraging in waters adjacent to the east coast of Queensland, Australia: three categories: Group A (FP tumours), Group B (non-tumoured skin from FP turtles) and Group C (non-tumoured skin from turtles without FP). Concurrent detection of ChHV5 and CmPV1 DNA is reported for all three categories, where Group A had the highest rate (43.5%). ChHV5 viral loads in Group A were significantly higher than loads seen in Group B and C. This was not the case for CmPV1 where the loads in Group B were highest, followed by Group A. However, the mean CmPV1 load for Group A samples was not significantly different to the mean load reported from Group B or C samples. Collectively, these results pivot the way we think about FP; as an infectious disease where two separate viruses may be at play.

## 1. Introduction

The endangered green turtle (*Chelonia mydas*) faces many threats, each of which need to be effectively managed in order to conserve this vulnerable species. Although the impact of disease in wild populations is largely unknown [1], green turtles are particularly susceptible to a tumour-forming disease [2]. Fibropapillomatosis (FP) is a neoplastic disease characterised by the formation of benign tumours. Fibrosarcomas, fibropapillomas, papillomas, fibromas and myxofibromas have all been associated with FP, with lesion type thought to be linked to different stages of development [3,4,5,6]. Depending on the location of growth, these tumours can impair feeding, vision, and locomotion of affected turtles [3,4,7]. Turtles with FP tumours are also more vulnerable to chronic stress and secondary infections [8,9]. Although this disease has been reported in all species of marine turtle, it predominantly affects green turtles [2].

While the aetiological agent of FP is yet to be confirmed [10], early studies provided evidence for a viral aetiological agent of FP [11,12]. A body of subsequent research has identified a range of potential candidates; this includes papillomaviruses, polyomaviruses, retroviruses and herpesviruses [4,13,14,15,16,17]. However, histological and molecular evidence has consistently linked FP to a herpesviral infection [11,12,14,15,18,19,20,21,22,23,24,25,26,27,28,29,30].

Chelonid alphaherpesvirus 5 (ChHV5) is considered to be the most likely aetiological agent of this disease. Elements of ChHV5 DNA have been amplified from FP tumours and from turtles without tumours, which is consistent with the latent nature of herpesviral infections [18,19,24,25,30,31,32]. However, despite the development of more sensitive assays, varying rates of ChHV5 detection in FP tumours have been reported [24,26,29,33,34,35]. It is possible that this variable rate of detection is the result of the different methods used in these studies. Alternatively, these results may simply be accurate for each population studied. It has been suggested that the inconsistency of ChHV5 detection in FP tumours indicates the presence of other co-factors contributing to disease manifestation [29].

Based on the morphology and histopathology of FP tumours, an alternative aetiological agent could be papillomavirus. The epidermal hyperplasia and mesenchymal proliferation observed in FP is similar to equine sarcoids, a neoplastic disease of horses caused by bovine papillomavirus type 1 or type 2 [36]. The characteristics of the dermal proliferation is also similar to those observed in cattle fibropapillomas and deer fibromas [12], which are both caused by papillomaviruses [37,38]. Moreover, transmission electron microscopy studies on samples from FP tumours have reported virions similar in morphology to papillomavirus [17]. These histopathological indicators triggered molecular investigations of a relationship between FP tumours and papillomaviruses a few decades ago, but they were not conclusive [17,39]. Subsequent research on links between papillomaviruses and FP were therefore abandoned until more recently, when a viral discovery project used primary cell lines from green turtles to screen a number of tissues from deceased turtles as well as FP tumour biopsies [40]. The cytopathic effect in cell cultures inoculated with material from the tumours was further investigated with current molecular techniques, which facilitated the identification of a papillomavirus. Mashkour et al. utilised species-specific assays for detection of virus [40], which likely contributed to the successful detection of both CmPV1 and ChHV5 in eight out of 10 FP tumour samples collected from green turtles with FP, thereby challenging the perception that FP is associated with just herpesvirus.

It is clear that the role of papillomaviruses in FP and the fact that viruses are the least studied pathogens in marine turtles [1] warrant further investigation. The overall objective of the current study was to conduct a comprehensive molecular survey to better understand the disease ecology. This objective was met through the following aims: (1) Investigate presence of ChHV5 and CmPV1 DNA in a larger sample collection from Australian turtles both with and without FP tumours and (2) Establish the viral load of ChHV5 and/or CmPV1 present in these samples.

## 2. Materials and Methods

### 2.1. Sample Collection

A total of 275 marine turtles were sampled at six locations along the Great Barrier Reef. This included 89 turtles with FP tumours, 87 of which were green turtles. The remaining turtles with FP tumours consisted of one loggerhead (*Caretta caretta*) turtles and one green/hawksbill (*Eretmochelys imbricata*) hybrid turtle. Where possible, paired samples were collected from FP tumours and non-tumoured skin. In addition, multiple samples of FP tumours from individual turtles were taken opportunistically. Samples from a further 186 non-tumoured turtles were also collected from three of the six study sites. These samples were grouped into three categories: Group A (FP Tumours), Group B (Non-tumoured skin from turtles with FP tumours) and Group C (Skin from non-tumoured turtles) (Table 1). The total dataset consisted of samples from waters adjacent to Cairns (*n* = 2), Townsville (*n* = 80), Bowen (*n* = 130), Airlie Beach (*n* = 1), Gladstone (*n* = 54) and Brisbane (*n* = 8).

Most turtles sampled in this study were captured using the rodeo capture technique [41] and flipper-tagged with a unique alpha-numeric inscribed titanium tag (Stockbrands Company, Pty. Ltd., Perth, Western Australia). The remaining tumour samples were collected during necropsy and others were donated (see Supplementary Tables S1–S3). The curved carapace length (CCL) of each turtle in this study was measured using a flexible tape measure (±2 mm).

All samples were collected using fresh, sterile, disposable scalpel blades and stored in cryovials containing 90% ethanol. Any paired skin samples were collected from non-tumoured sites on the trailing edge of the front flipper of each turtle. Samples were collected between 2009 and 2018. The median year of collection was 2013. All samples were stored at 4°C prior to DNA extraction. All live turtles were sampled under permits from James Cook University Animal Ethics Committee (A1501 and A1971), Department of Environment and Science (WISP06619309 and WISP13754613) and Great Barrier Reef Marine Park Authority (G12/35326.1, G10/33220.1 and G36593.1).

### 2.2. DNA Extraction

DNA was extracted using Promega Wizard1 SV Genomic DNA Purification System according to the manufacturer’s instructions with an additional 10 μL of proteinase K used per reaction. DNA concentration was assessed by spectrophotometric analysis (NanoDrop™ 2000 Spectrophotometer), and DNA purity was determined using OD_260/280_. Extracted DNA was stored in −20 °C prior to analysis. All samples containing ≥2ng/µL of DNA were used for subsequent analysis.

### 2.3. Primer and Probe Design

PCR primers and probes were designed and used to specifically amplify conserved regions of CmPV1 and ChHV5: the E1 gene and DNA polymerase, respectively. The green turtle Glyceraldehyde 3-phosphate dehydrogenase (GAPDH) gene was selected to quantify a single-copy host target as a proxy to host cell copy number. These targets will hereby be referred to as CmPV1-E1, ChHV5-DNApol and GAPDH. In addition, one set of primers was designed to target the E2 gene from CcPV1 from loggerhead turtles (CcPV1-E2).

Primers and/or probes for ChHV5-DNApol and GAPDH targets were designed in this study, while CmPV1-E1 target was amplified using the Cm-Pap-109 primer and probe set described in Mashkour et al. (2018) (Table 2). Primer and probe design for the remaining targets was based on sequences available from GenBank (National Center for Biotechnology Information; NCBI, Bethesda, Maryland). Accession numbers HQ878327.2 and FJ234450.1 served as the main base for the ChHV5-DNApol and GAPDH primers respectively. To increase the specificity of the assay, ChHV5-specific primers were designed based on the consensus sequences of DNA polymerase sequences isolated from green turtles only. Cc-Pap-99 primer set was designed to specifically target CcPV1-E2 gene. The E2 genes of CmPV1 (Genbank accession no. EU493091.1) and CcPV1 (GenBank accession no EU493092.1) were aligned to identify the site of highest variability. This section of the CcPV1-E2 was then used to design specific PCR primers to detect only CcPV1. All primers and probes were designed using AlleleID version 7.7 (Premier Biosoft International, Palo Alto, California) and synthesised by Macorgen Inc. (Seoul, South Korea) (Table 2).

Probe-based qPCR reactions (20 μL) were made with 10 μL of GoTaq^®^ Probe qPCR Master Mix (Promega, Madison, WI, USA), 1.6 μL of each primer (0.8 μM), 0.2 μL of probe, ~2 μL of template DNA (~80 ng) and 4.6 μL nuclease free water. All GoTaq^®^ Probe qPCRs were carried out using a Magnetic Induction Cycler (MIC) qPCR machine (Bio Molecular Systems). The thermocycling conditions for these assays were as follows: 2 min at 95 °C followed by 50 cycles (95 °C for 10 s, 60 °C for 10 s). All assays without a probe were run as dye-based qPCR reactions (20 μL) which were comprised of 10 μL of 5× GoTaq^®^ qPCR Hot Start Master Mix (Promega), 1.6 μL of each primer (0.8 μM), ~2 μL of template DNA (~80 ng) and 4.8 μL nuclease free water. The PCR was carried out on a Rotor-Gene 6000 thermocycler machine. The results of these assays were visualised in real time with the thermocycling conditions provided in Table 1.

A green turtle embryo fibroblast cell line, CMEM which previously tested negative for herpesviral and papillomaviral DNA using published PCR primers (Mashkour et al., 2018), was used as the negative biological control for these assays.

### 2.4. Producing Standard Curves for ChHV5, CmPV1 and Green Turtle Genomic DNA

To be able to report viral copy numbers, robust standards were required from each of the viruses and the host samples. For ChHV5-DNApol and GAPDH targets, Cm-Dpol-864 and GAPDH-475 assays were used to generate amplicons for use as plasmid inserts (Table 2). At the time, the Australian CmPV1 strain was not fully sequenced and to avoid possible SNPs and missing samples, for CmPV1-E1 target the Cm-Pap-109 primers were used from Mashkour et al. [40].

After successful polymerisation, the amplicons CmPV1-E1, ChHV5-DNApol and GAPDH were cloned separately into pGEM^®^-T Easy Vectors (Promega, Madison, WI, USA) using the TA blue/white cloning system according to pGEM^®^-T Easy Vector Systems protocol. To confirm the presence of desired inserts the plasmid DNAs were sequenced along both strands using a universal M13 primer set (Macorgen Inc., Seoul, Korea).

A supercoiled circular plasmid can suppress the PCR amplification and result in an error as big as seven copies of the target gene per cell [42]. To avoid misinterpretation of viral loads the cloned plasmids were treated with restriction enzymes. To select an enzyme which only cut the pGEM^®^-T Easy Vector once, without digesting the target genes, the vector and the cloned sequences were analysed via Geneious v10.1 [43], subsequently SacI and PstI were chosen for treatment. The linear plasmids were eluted in DNAse free water and stored at −20 °C.

### 2.5. Calibration Curves and Real-Time Quantitative PCR

For the setup of a dilution series, the mass of one copy of the cloned CmPV1-E1, ChHV5-DNApol and GAPDH DNA plasmid were calculated using the mass of 1bp. The gram per mol of each amplicon was used to determine the appropriate amount needed for the initial dilution steps. The measurement was obtained using a Quantus™ Fluorometer using QuantiFluor^®^ dsDNA Systems (both from Promega, Madison, WI, USA).

Tenfold serial dilutions were prepared in nuclease free water to produce a titration series from 10^8^ to 10^1^ of each plasmid in 2 μL/reaction (as the GoTaq^®^ Probe qPCR was undertaken using 2 μL of template DNA in a 20 μL PCR). From this, calibration curves for pCmPV1-E1, pChHV5-DNApol and pGAPDH were produced. To make the standard curves, each dilution point was assayed in triplicate and the highest efficiencies were considered for the following calculations. Calibration curves for pCmPV1-E1, pChHV5-DNApol and pGAPDH were constructed by plotting cycle numbers versus the log concentrations using the MIC software (see Figure S1).

### 2.6. Viral and Host qPCR Reaction Conditions and Data Interpretation

Linear pCmPV1-E1, pChHV5-DNApol and pGAPDH were run independently in singleplex qPCR reactions. Samples were tested in duplicates and in each run a negative biological control (green turtle primary culture), a no template control (NTC; nuclease free water) and triplicate standards were included. The MIQE guidelines [44] were followed to avoid assay contamination by genomic or plasmid DNA. To ensure repeatability, a single person ran the qPCRs and measures of intra- and inter-assay variation were used. Care was taken to provide a wide range standard curve and cover the copy number range observed in “*unknown*” samples. Samples were considered positive if the emitted fluorescence of both duplicates exceeded the threshold limit of detection (LOD) and were within 0.5 C_t_ of each other and negative otherwise. Lower LOD was determined by carrying out qPCR on 24 replicates of low copy number per reaction for each plasmid (5, 10, 50 and 100 target copies per 2 μL). The 50% and 100% LOD were calculated for linear pCmPV1-E1, pChHV5-DNApol and pGAPDH.

The runs were considered valid if the efficiency was between 90–110%, NTC and negative control were negative and if the MIC standardised value would let import the normalised standard curve for the run. Data were analysed in the MIC software where an adaptive baseline threshold was automatically generated for each assay. PCR efficiencies were calculated using the formula: E = (10^−1/slope^ − 1) × 100.

The viral copy number of each virus per cell was calculated according to CmPV1-E1 and/or ChHV5-DNApol copies per host cell using the following equation:

(1) $$=\frac{\mathrm{CmPVE}1\;\mathrm{and}/\mathrm{or}\;\mathrm{ChHV}5\mathrm{DNApol}\;\mathrm{quantity}}{\;\frac{1}{2}GAPDH\;quantity}$$

For all samples, viral load per diploid genome was determined by dividing CmPV1-E1 and ChHV5-DNApol copy numbers by half of the GAPDH copy number.

### 2.7. Statistical Analysis

The resulting copy numbers of group A and B were compared using unpaired t-test with a 95% confidence interval. The two-tailed *p*-value was also calculated. Dealing with non-parametric groups of results, the Kruskal–Wallis test by ranks was done on samples from different regions to investigate the distribution of each virus in six regions covered by this study. The rate of detection was calculated using Chi-square and Fisher’s exact test of independence and was used to compare the portion of animals, tumours, non-tumoured skin from turtles with FP tumours, and skin from non-tumoured turtles, with each virus in each region. Values with *p*-value < 0.05 were considered statistically significant. The statistical analyses were conducted using IBM SPSS Statistics 25. The mean copy number of ChHV5 in each sample group in the present study was also compared with results in other ChHV5 studies [25,45,46]. Unit conversions were done where appropriate.

## 3. Results

### 3.1. qPCR Validation and Optimisation

In silico evaluation of the designed primers and probes confirmed the specificity of these oligonucleotides. Initial amplified products and plasmid amplicons were sequenced and had 100% pairwise identity to the desired target genes. The known negative controls (both NTC and biological control) did not react in any assay.

The standard curves were successfully constructed and used for the qPCRs and subsequently the calculation of copy numbers per cells of each virus. The standard curves for linear pGAPDH, pCmPV1 and pChHV5 are shown in supplements (Figure S1). Absolute quantification and the standard curves for three cloned plasmids were plotted based on the cycles (Cq values) and the log of concentration (10 to 10^8^ copies per reaction). The data points represent three replicates of each dilution. The R^2^ values, the efficiency of each qPCR and the equation of the standard curves are as follows: pGAPDH R^2^ 0.950; Efficiency 0.99; Equation y = −3.35x + 40.76; pChHV5 Dpol R^2^ 0.993; Efficiency 0.92; Equation y = −3.54x + 40.49; pCmPV1-E1 R^2^ 0.974; Efficiency 1.05; Equation y = −3.21x + 40.48.

The qPCR sensitivity was determined by 50% certainty cut-off for LOD as described by World Organization for Animal Health [47]. In this study, 10 copies per reaction were detected in 75% of the replicates for CmPV1-E1 and ChHV5-DNApol while 50 copies per reaction were detected for GAPDH. The 100% LOD was 100 copies per reaction for all the three plasmid DNAs.

### 3.2. Viral DNA Detection

A total of 353 samples from 275 foraging turtles were screened in this study using probe-based qPCR. Of the turtles sampled in this study, 89 individual turtles were afflicted with FP tumours. On an individual turtle basis, the detection of CmPV1 and/or ChHV5 amplicons varied from 0 to 100% in turtles with FP tumours. If one sample from one animal reacted in the PCR, the animal was considered positive for the presence of the tested virus. Of these 89 turtles, 69 (77.3%) tested positive for ChHV5, 46 (51.7%) for CmPV1, 40 (44.9%) for both viruses and samples from 14 (15.7%) turtles tested negative for both. Only 6 (6.7%) turtles were positive for CmPV1 but not ChHV5, while 29 (32.6%) turtles were positive for ChHV5 but not CmPV1.

Viral DNA detection of both ChHV5 and CmPV1 varied between sample types. Group A had the highest rates of viral detection and within this group ChHV5 was detected in 86.3% of samples (Table 3). This is almost double the rate of detection of CmPV1 (47.3%) in that sample type. Moreover, 57 of the 62 samples in which CmPV1 was detected also reacted to the ChHV5 assay. Therefore, ChHV5 DNA was more predominant in FP tumours but was often detected alongside CmPV1. Only 9.2% of the Group A samples did not react in either assay (Table 3). Interestingly, a higher rate of detection for both viruses was observed in the Group C samples compared to Group B, despite the Group C samples being sourced from turtles without FP tumours (Table 3). Similarly, of the samples from which no viral DNA was detected Group B samples had the highest rate (Table 3).

Using the specific primer sets for loggerhead papillomavirus (Cc-Pap-99 PCR primers), CcPV1 was detected in both samples of loggerhead turtles tested, one of which was taken from tumour tissue (Group A) and the other was a Group B sample. The tumour tissues of loggerhead samples did not react in CmPV1-specific assays and the green samples did not react in CcPV1-specific assays. This provides strong support for the already defined species barrier between the CmPV1 and CcPV1 viruses.

#### Viral Loads of CmPV1 and ChHV5

The mean viral loads detected in each of the three sampling groups are reported in Table 4. The ChHV5 load in Group A ranged from 0 to 226.1 (mean: 15.635; SD: 30.152) copies in each cell and the CmPV1 load range was 0 to 2.54 (mean: 0.0814 (≈1 copy in 12 cells); SD: 0.357). The raw data is provided in Supplementary Tables S1–S3. With respect to ChHV5, the highest viral load was detected in the Group A samples (FP tumours). Interestingly, the mean ChHV5 load for Group C samples was slightly higher than that for Group B despite the fact that Group B samples were collected from turtles afflicted with FP. The viral load per cell of ChHV5 in Group A samples is significantly higher than Group B samples (*p*-value < 0.0001). The mean viral load of CmPV1 varied amongst the sample groups, with the highest load detected in Group B (Table 4). The FP tumour samples (Group A) had a lower mean load of CmPV1 than Group B, this difference is statistically significant (*p*-value = 0.0138). Overall, the highest mean viral load detected was ChHV5 in Group A samples (Table 4).

The mean copy number of ChHV5 in each sample group in the present study was found to be consistent with results in other ChHV5 studies (See Supplementary Table S4). No similar undertaking could be done for the CmPV1 results as there are no comparable datasets in marine turtles. However, the results are consistent with human cervical intraepithelial neoplasia (CIN) I, II, III caused by human papillomavirus (HPV) 16, 18, 33 and 58 [48,49,50].

### 3.3. Geographic Distribution of ChHV5 and CmPV1

**Figure 1:**
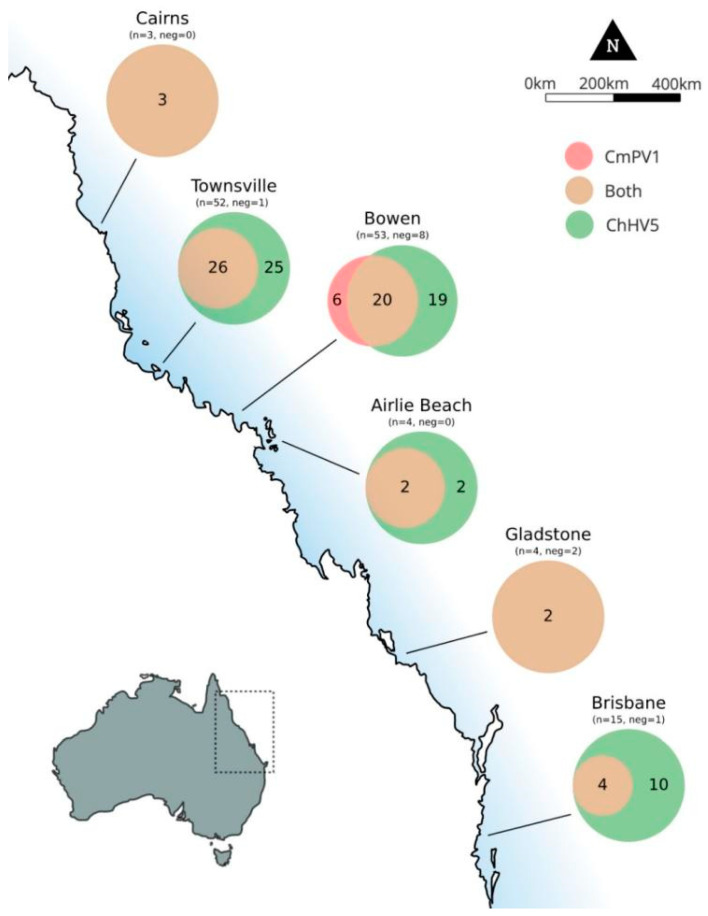
The distribution of chelonid alphaherpesvirus 5 (ChHV5) and *Chelonia mydas* papillomavirus 1 (CmPV1) in FP tumour samples (Group A) collected from green turtles at six regions along the Queensland coast. A total of 131 FP tumour tissues were tested. Of the assay samples 62 reacted in the qPCR for CmPV1, 113 for ChHV5, and 12 samples did not react in either assay. 57 turmor samples reacted in both viruses. The viral detection at each site is categorised into three categories: ChHV5 only (red), CmPV1 only (green), both ChHV5 and CmPV1 detected in the same sample (beige). The total number of samples screened (n) and the total number of negatives (neg) are shown for each location.

Viral detection varied both between the six regions in this study, and within regions but between viruses (Figure 1). In Group A samples from both Townsville, Brisbane, and Bowen, ChHV5 was detected in significantly higher rates than CmPV1 (*p* < 0.05) (Table 5). This is also true of Group C samples from Gladstone (*p* < 0.001) (Table 5). However, in Group C samples collected from Bowen, significantly higher rates of CmPV1 were detected (*p* < 0.001) (Table 5).

#### Geographic Distribution of Viral Loads

**Figure 2:**
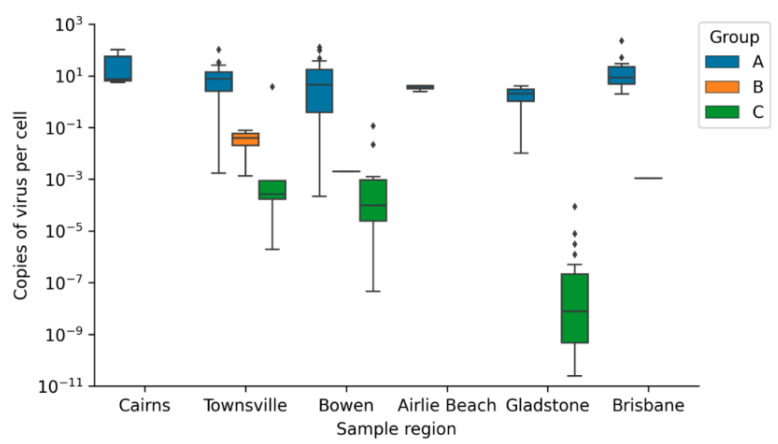
Boxplots of ChHV5 viral loads (log scale) in different sample types from six regions of Queensland. Group A (FP Tumours), Group B (Non-tumoured skin from turtles with FP tumours) and Group C (Skin from non-tumoured turtles). The Box plots of viral loads include the median (centre horizontal line), interquartile range (box), minimum and maximum (whiskers), and outliers (diamonds).

The distribution of ChHV5 viral load is comparable in Group A samples across the six regions tested, as Kruskal–Wallis test by ranks resulted in a non-significant difference between the ChHV5 viral load in tumour samples from these six regions (ChHV5_GroupA_ *p* value = 0.100). Group C turtles from these regions also do not have significantly different loads of ChHV5 (ChHV5_GroupC_ *p* value = 0.122). The viral load distributions in samples that contained ChHV5 DNA from six regions of Queensland can be seen in Figure 2.

**Figure 3:**
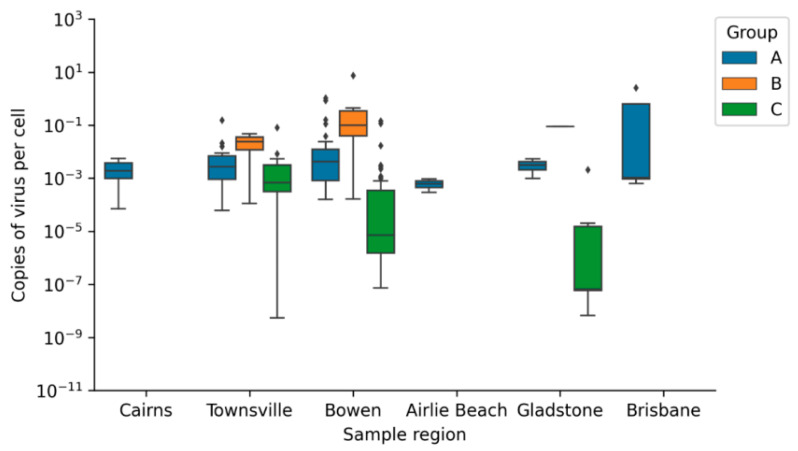
Box-plots of CmPV1 viral loads (log scale) in different sample types from six regions of Queensland. Group A (FP Tumours), Group B (Non-tumoured skin from turtles with FP tumours) and Group C (Skin from non-tumoured turtles). The Box plots of viral loads include the median (centre horizontal line), interquartile range (box), minimum and maximum (whiskers), and outliers (diamonds).

The distribution of CmPV1 load is the same in the tumours sampled across the six regions tested, as Kruskal–Wallis test by ranks resulted in a non-significant difference between the CmPV1 viral load in tumour samples from these six regions (CmPV1_GroupA_ *p* value = 0.317). This trend can be seen in Figure 3.

## 4. Discussion

Although papillomavirus was proposed as an aetiological agent of FP in the 1990s [4] and this hypothesis was supported by transmission electron microscopy evidence [17], papillomaviral DNA was not successfully detected in FP tumour tissue in studies conducted around this time [17,39]. Conversely, a body of histological and molecular evidence emerged which led to herpesviral infection to be considered the primary candidate for an aetiological agent of FP [11,12,14,15,19,20,21,22,23,24,25,26,27,28,29,30,33]. Despite the subsequent discovery and description of CmPV1 and CcPV1 [51,52], these papillomaviruses were not linked to FP lesions. However, following the initial detection of both ChHV5 and CmPV1 DNA in a small sample size of FP tumour tissue [40], the current study set out to test the wider prevalence of CmPV1 and co-occurrence with ChHV5 in marine turtles in waters adjacent to the east coast of Queensland, Australia. Three sample categories were used in this study: Group A (FP tumours), Group B (non-tumoured skin from turtles with FP tumours) and Group C (non-tumoured skin from turtles without FP tumours). Concurrent detection of ChHV5 and CmPV1 DNA is reported for all three categories which challenges previous hypotheses of ChHV5 as the leading etiological agent of FP. This study is the first to report concurrent detection of viral DNA from ChHV5 and CmPV1 in non-tumoured skin samples collected from marine turtles. This study is also the first comprehensive molecular survey of CmPV1 utilising species-specific primers, allowing for a more in-depth investigation of the results in Mashkour et al. [40]. However, the results of this study also provide further evidence toward the association between ChHV5 and FP, where higher detection rates of ChHV5, compared to CmPV1, are reported in FP tumour samples (Group A). Furthermore, regional differences in the rate of detection and viral loads in Group A samples support the hypothesis of FP tumour “hot-spots” in Queensland [53]. Collectively, these results pivot the way we think about FP; as an infectious disease where two separate viruses may be at play.

### 4.1. Rate of Detection

Overall, the rate of detection for ChHV5, CmPV1 and the combination of both was highest for Group A (Table 3). These results are consistent with the theory that ChHV5 is associated with FP tumours [2,11,15,19,25], and open a new line of scientific inquiry regarding the role of CmPV1 in FP tumour development. However, if a simple cause-and-effect relationship is at play, a detection rate of 100% in FP tumour samples would provide strong support of this. Although the rates of detection of these viruses were highest in FP tumour samples, these rates were still variable; ChHV5 was detected in 86.3% of FP tumour samples, while CmPV1 was detected in less than half (47.3%) of these samples. Although there is no comparable data available for CmPV1, this variable rate of ChHV5 detection is consistent with similar studies [18,24,26,29,34]. However, the detection of CmPV1 in Group A samples provides yet another small piece in the puzzle for the trigger factors involved in FP tumour development. Taken together, these results add weight to the theory that there are other factors at play in FP development [2,29,54].

This study also serves as a validation for newly developed qPCR assays for the detection of ChHV5, CcPV1 and GAPDH described herein. Of note is the improved sensitivity of the Dpol-ChHV5-82 assay in comparison to conventional PCR assays previously used in this region by Jones et al. [29] to facilitate longer amplicon length for subsequent phylogenetic analyses. Using a combination of conventional PCR assays ChHV5 DNA was detected in 62 of the 89 Group A individual turtles [29]. In the present study, ChHV5 DNA was detected in 69 Group A turtles using a single qPCR assay (Dpol-ChHV5-82), which emphasises the need to match the assay selection with end-point goals. Consistent with the known advantages of qPCR [23,40,55], our results show that qPCR assays are suitable in studies which aim to determine presence or absence of viral DNA, due to the enhanced sensitivity of these assays over conventional PCR. Moreover, the benefits of qPCR assays can extend beyond simple presence/absence tests into viral load assessment for positive samples.

### 4.2. Mean Viral Load

Here, we reported viral loads for both ChHV5 and CmPV1 across three sample types (Groups A, B and C). The highest mean load of ChHV5 was found in Group A samples, adding to the body of evidence that this virus is closely associated with FP tumours. The highest mean load of CmPV1 was detected in Group B samples, but the significance of this result is unclear. The small sample size comprising this Group (*n* = 36), in contrast to Group A and C (*n* = 131 and 186 respectively), limit the conclusions that can be drawn about this result. Further analysis on a larger set of samples of this type (non-tumoured skin from turtles with FP tumours) would undoubtedly add resolution to this result. Other than the case of ChHV5 in Group A samples, the mean viral load in all other sample types for both viruses was less than one copy per cell indicating a low level infection in these samples.

While the mean ChHV5 load results obtained in this study were consistent with other reports [23,25,45,46], such comparisons could not be made for CmPV1 as there are no other comparable datasets in marine turtles. However, the CmPV1 viral loads are in the same range reported for human cervical malignancies caused by HPV 16, 18, 33 and 58 where the mean HPV copies per 10,000 cells were reported to be ~log3–5 in CIN I, II and III [48,49,50].

This study assessed both the rate of detection and viral load of ChHV5 and CmPV1 and, in some cases, there was disparity between the rate of detection and viral load. For example, CmPV1 was detected in 100% of Group A samples from Cairns yet the viral load was relatively low (0.002 ± 0.002 copies per cell). This phenomenon may be a feature of the varied storage times prior to extraction and qPCR; while all samples were stored at the same temperatures some were stored for 10 years prior to analysis, while others were stored for less than one. It is possible that this led to DNA degradation, with subsequent impacts on viral load detection. However, these results are in comparison to a ChHV5 load of 39.300 ± 56.788 copies per cell in the same samples with the same rate of detection. Such marked variation in viral load in the same sample (subject to the same storage time) adds to the complexity of the picture, rather than a presence/absence result.

Researchers have detected and quantified ChHV5 through molecular surveys and serological analyses in tumoured and non-tumoured marine turtles [23,25,32,45,46,56,57]. Whole genome sequencing and transcriptome approaches have also been employed in recent years to better understand the association between ChHV5 and FP [58,59,60]. However, additional molecular and serological surveys specifically targeting both ChHV5 and CmPV1 from other regions and species of marine turtles would be highly beneficial to understanding the role of CmPV1 in FP, and in marine turtle health as a whole.

### 4.3. Concurrent Detection

Concurrent infections of herpesvirus and papillomavirus have been described extensively in humans (see [61]) and in Atlantic bottlenose dolphins [62]. Our study reports the concurrent presence of herpesviral and papillomaviral DNA in both tumoured and non-tumoured marine turtles. The presence of these viruses in non-tumoured skin of turtles with FP tumours (Group B) and those free of clinical disease (Group C) are consistent with previous studies on these viruses [23,46,63] and are in line with the ubiquity and commensal nature of both viruses [46,64]. These results lend further weight to the hypothesis that FP manifestation relies on more than just exposure to viral infection alone [2].

Due to the nature of these two viruses, there is a possibility that either ChHV5 or CmPV1, or indeed both may be opportunistic pathogens in FP, rather than aetiological agents. As Koch’s postulates have yet to be fulfilled for either virus, this possibility needs to be considered when interpreting the results of this study. Future studies, considering ChHV5 and/or CmPV1, or even other emerging viruses, may aid in elucidating the answer to this question.

### 4.4. Geographic Locations

Differences in both viral load and rate of detection of both ChHV5 and CmPV1 were found across the six study sites. The highest mean viral load was detected in Cairns. This group was comprised of three samples from two turtles which were both afflicted by FP tumours. Higher rates of detection and viral loads are reported in Group A samples from Townsville, Bowen and Brisbane. These results are consistent with previous suggestions that these regions are FP “hot-spots” in Queensland [53]. However, when examining the data beyond mean loads, it is clear that higher loads of ChHV5 were detected in Group A samples at each study site. This suggests that while there may indeed be FP tumour hot-spots in Queensland, this relationship is not directly linked with viral load. Bowen is of particular interest, being the only study site where some Group A samples contained CmPV1 exclusively. In all other study sites, when CmPV1 was detected from Group A samples it was in conjunction with ChHV5. Moreover, Group C samples from Bowen had a higher rate of CmPV1 detection compared to ChHV5 (*p*-value < 0.001) indicating a high prevalence of papillomavirus in clinically healthy turtles in this region, which would make it an excellent study site for more in-depth investigations on CmPV1.

Although the migratory nature of marine turtles should be considered when interpreting these results, only fine-scale movements have been observed between foraging grounds in Queensland [65]. This high site fidelity adds increased confidence in the locational trends reported here. However, it is possible that moribund turtles alter their foraging and site-selection behaviours during illness. Long-term monitoring programs which document and sample FP cases would provide a robust means to ensure that capture location is accurately reflecting a well-defined foraging ground.

### 4.5. Limitations

In a field that relies on opportunistic sampling of free ranging marine wildlife, it was a major effort to collect samples (*n* = 353) from a total of 275 individual turtles, especially those with FP. Although more samples distributed evenly between sample groups and study sites may have provided more confidence in the conclusions that can be drawn regarding these categories, such sample sets are not always realistic in wildlife disease investigations [29]. Therefore, in the interest of elucidating the potential role of papillomavirus in FP as identified by Mashkour et al. [40], we have reported the findings for all sites and sample types. Testing the tissue samples from rehabilitation facilities or existing biobanks can be an alternative to assessing free range turtles and is therefore suggested to test for CmPV1 presence in FP tumours from numerous global locations.

Tumour severity scores, tumour size and anatomical location are not available for most samples utilised in this study. This precluded further analysis concerning these factors. We are therefore unable to ascertain whether there are any correlations between viral load and tumour size, location or severity of FP affliction. Future studies should consider these factors in order to provide deeper insight to the trends reported here.

Assay sensitivity and specificity is always a consideration and as tests are constantly evolving, future development may include more sensitive methods and targets such as viral mRNA as an indicator of viral expression. However, ChHV5 mRNA has been observed to be less abundant than DNA, with most viral genes not being highly expressed in FP [58,59,66]. Moreover, no viruses, including ChHV5 and CmPV1, were found to be differentially expressed in FP tumours when de novo transcriptome assembly was employed [66]. While these results indicate that future viral expression studies on FP tumours could encounter challenges in establishing differential expression, rapid advancements in molecular biology may aid in overcoming these challenges. As such, existing FP RNA-seq datasets should still be specifically queried for CmPV1 expression and further studies on viral expression should be encouraged. In the present study, we reported low viral copy numbers demanding sensitive assays. To overcome assay sensitivity targeted sequencing, droplet digital PCR, and low input template DNA qPCR-based approaches such as amplification ratio-based detection can be implemented. The latter was used in marine turtle population genetics and FP related eDNA analysis [59,67].

### 4.6. Future Perspective

Despite the noted limitations, the results reported in the present study show some clear and reliable trends which we hope will be further explored on a global scale. Our understanding of FP, and marine turtle virology as a whole, has many knowledge gaps [1] which need to be addressed in order to develop comprehensive management plans for these endangered species. The results from this and other studies have revealed that viral DNA is present in animals that do not show clinical signs [18,56,58], which questions their role in tumour development and highlights the need for further understanding of co-factors (environmental triggers, host genetics and immune responses) in disease development and why some turtles do not develop tumours despite the presence of viral DNA. Diseases in terrestrial animals are often tracked by serological methods, and such tests could be more widely implemented to determine whether turtles have mounted an immune response to ChHV5, CmPV1 and/or CcPV1 which would negate the need to rely on clinical signs alone for exposure status of an animal [9]. Recently, Page-Karjian et al. screened blood samples from a large group of free ranging and rehabilitated green and loggerhead turtles. All samples were analysed for ChHV5 DNA using qPCR, and turtles in rehabilitation for antibodies to ChHV5 peptides using an enzyme-linked immunosorbent assay (ELISA). A higher proportion of loggerheads were found to be ChHV5-positive using ELISA, suggesting the hypothesis that they may be more able to mount an immune response against the virus when compared to green turtles [56]. We were unable to collect corresponding blood samples from FP-afflicted turtles in this study, precluding such analyses. Our understanding of FP would be greatly improved if such studies were employed across a range of species and regions.

In terms of CmPV1 in FP tumours, the presence, infectivity and tumorigenesis should be assessed by advanced virological methods such as transmission electron microscopy, serology, histopathology, cell culture and molecular analyses. To study the virus itself, molecular analyses can be further applied to map any evolutionary variations in each region.

### 4.7. Conclusions

The results of the present study provide valuable insight into some of these gaps and with improved detection of papillomavirus in FP tumour tissue, it also challenges long held perceptions of the role of herpesvirus as the sole infectious agent associated with FP tumours. This study serves as an open door to broaden our horizon when surveying FP- afflicted turtles.

## Figures and Tables

**Table 1:** The number of turtles used in this study categorised by study site and sample type.

			Number of Samples		
Study Site	Total Number of Turtles	Number of Turtles with FP Tumours	Group A (FP Tumours)	Group B (Non-Tumoured Skin from Turtles with FP Tumours)	Group C (Skin from Non-Tumoured Turtles)
Cairns	2	2	3	-	-
Townsville	80	24	52	13	56
Bowen	130	50	53	15	80
Airlie Beach	1	1	4	-	-
Gladstone	54	4	4	4	50
Brisbane	8	8	15	4	-
Total	275	89	131	36	186

**Table 2:** The sequences of the PCR primers and probes along with the PCR cycling protocols used in the PCR to amplify the target genes from cloned plasmids (standards), positive controls and test samples. Abbreviations: BHQ1, black hole quencher-1; FAM, 6-carboxyfluorescein; F, forward primer; R, reverse primer; P, probe.

	Primers and Probes	Sequence	Target Gene	Amplicon Length	Cycling Conditions	Reference
Cloned plasmids for standard assays	Cm-Dpol-864	F: 5′-ATG ACG GAC GGA CAA CAG-3′	ChHV5-DNApol	864 bp	2 min at 95 °C; 35 cycles (95 °C for 10 s, 57 °C for 15 s, and 72 °C for 55 s)	The present study
		R: 5′-GGA GAT GAC GGC TGC TAA-3′				
	GAPDH-475	F: 5′-CCT TTA ATG CGG GTG CTG-3′	GAPDH	475 bp	2 min at 95 °C; 35 cycles (95 °C for 10 s, 57 °C for 15 s, and 72 °C for 30 s)	The present study
		R: 5′-CAC GGT TGC TGT ATC CAA-3′				
	Cm-Pap-109	F: 5′-GCC GAT GAT GTC CAC TTA T-3′	CmPV1-E1	109 bp	2 min at 95 °C; 40 cycles (95 °C for 10 s, 60 °C for 20 s, and 72 °C for 30 s)	Mashkour et al. (2018)
		R: 5′-GCT GAA TCC ACA GAG GTA G-3′				
Screening assays	Cm-Pap-109	F: 5′-GCC GAT GAT GTC CAC TTA T-3′	CmPV1-E1	109 bp	2 min at 95 °C; 50 cycles (95 °C for 10 s, 60 °C for 10 s)	Mashkour et al. (2018)
		R: 5′-GCT GAA TCC ACA GAG GTA G-3′				
		P: 5′-FAM CGA CCC ATG AAG CCG CTG T BHQ1-3′				
	Dpol-ChHV5-82	F: 5′-CTA CCT TGT CTG GAG GTG GC-3′	ChHV5-DNApol	82 bp	2 min at 95 °C; 50 cycles (95 °C for 10 s, 60 °C for 10 s)	The present study
		R: 5′-GGG TGT GAA TAA AAT CCC GCG-3′				
		P: 5′-FAM TAG GGC GCG ACA TGC TTC BHQ1-3′				
	GAPDH-83	F: 5′-CTG GTC TCC TGG TAT GGA-3′	GAPDH	83 bp	2 min at 95 °C; 50 cycles (95 °C for 10 s, 60 °C for 10 s)	The present study
		R: 5′-CAT GGA CTC CCA ACC TAT C-3′				
		P: 5′-FAM AAA CCA CCC TCC AAA TCT GGC BHQ1-3′				
	Cc-Pap-99	F: 5′-AAA GGG CAG TGG GAA ATC TC-3′	CcPV1-E2	99 bp	2 min at 95 °C; 40 cycles (95 °C for 10 s, 58 °C for 20 s, and 72 °C for 30 s)	The present study
		R: 5′-TGT GAT GGC GAC GAT GTG-3′				

**Table 3:** The results from the 353 samples screened in this study for the presence of chelonid alphaherpesvirus 5 (ChHV5) and *Chelonia mydas* papillomavirus 1 (CmPV1). The results are categorised by sample types: Group A (FP Tumours), Group B (Non-tumoured skin from turtles with FP tumours) and Group C (Skin from non-tumoured turtles). A summary of the total detections is also provided.

	Viral DNA Detections			
Sample Type	ChHV5	CmPV1	Both ChHV5 and CmPV1	Neither ChHV5 and CmPV1
Group A	113/131 (86.3%)	62/131 (47.3%)	57/131 (43.5%)	12/131 (9.2%)
Group B	4/36 (11.1%)	9/36 (25.0%)	1/36 (2.8%)	24/36 (66.7%)
Group C	39/186 (21.0%)	68/186 (36.6%)	8/186 (4.3%)	85/186 (45.7%)
Overall	156/353 (44.2%)	139/353 (39.4%)	66/353 (18.7%)	121/353 (34.3%)

**Table 4:** Mean viral load (reported as copy number per cell ± standard deviation) detected in the three different biological sample types screened in this study; Group A (FP Tumours), Group B (Non-tumoured skin from turtles with FP tumours) and Group C (Skin from non-tumoured turtles).

	ChHV5 (Copy Number Per Cell)	CmPV1 (Copy Number Per Cell)
Group A	15.635 ± 30.152	0.0814 ± 0.357
Group B	0.0204 ± 0.0379	0.855 ± 2.351
Group C	0.100 ± 0.606	0.005 ± 0.023

**Table 5:** Number of viral DNA detections and mean viral load (reported as copy number per cell ± standard deviation) of the samples which tested positive in the three different biological sample types screened in this study; Group A (FP Tumours), Group B (non-tumoured skin from turtles with FP tumours) and Group C (skin from non-tumoured turtles). The number of detections within each group were compared using Chi square and Fisher’s exact test, respectively, with the results also reported here. Statistically significant differences are highlighted in bold. Unavailable samples are denoted by a hyphen. * This is not a meaningful comparison.

		Group A			Group B			Group C		
		ChHV5	CmPV1	*p*-Value	ChHV5	CmPV1	*p*-Value	ChHV5	CmPV1	*p*-Value
Cairns	*No. (%) positive*	3/3 (100.0%)	3/3 (100.0%)	1.000	-	-	-	-	-	-
	Average copy number/cell	39.300 ± 56.788	0.002 ± 0.002	*	-	-	-	-	-	*
Townsville	*No. (%) positive*	51/52 (98.1%)	26/52 (50.0%)	**<0.00001**	2/13 (15.4%)	2/13 (15.4%)	1.000	5/56 (8.9%)	11/56 (19.6%)	0.105
	*Average copy number/cell*	11.364 ± 16.120	0.010 ± 0.030	*	0.039 ± 0.053	0.023 ± 0.033		0.758 ± 1.695	0.009 ± 0.024	*
Bowen	*No. (%) positive*	39/53 (73.6%)	26/53 (49.1%)	0.0095	1/15 (6.7%)	6/15 (40.0%)	0.0309	17/80 (21.25%)	52/80 (65%)	**<0.00001**
	Average copy number/cell	16.456 ± 28.911	0.088 ± 0.257	**-**	-			0.231 ± 0.918	0.005 ± 0.024	*
Airlie Beach	*No. (%) positive*	4/4 (100%)	2/4 (50.0%)	0.429	-	-	-	-	-	-
	Average copy number/cell	3.554 ± 0.835	0.001 ± 0.0004	*	-	-	-	-	-	*
Gladstone	*No. (%) positive*	2/4 (50.0%)	2/4 (50.0%)	1.000	0/4 (0.0%)	1/4 (25.0%)	1.000	22/50 (44.0%)	6/50 (12.0%)	**0.0003**
	Average copy number/cell	2.061 ± 2.900	0.003 ± 0.003	*	-	-	-	0.000004 ± 0.00001	0.0003 ± 0.0008	*
Brisbane	*No. (%) positive*	14/15 (93.3%)	4/15 (26.6%)	**<0.001**	1/4 (25.0%)	0/4 (0.0%)	1.000	-	-	-
	*Average copy number/cell*	29.220 ± 58.466	0.634 ± 1.267	*	-	-	-	-	-	*

## Data Availability

All data presented in this study are available herein.

## Supplementary Materials

The following are available online at https://www.mdpi.com/2076-2615/11/3/697/s1, Figure S1: Absolute quantification and the standard curves for three cloned plasmids, Table S1: The origin of FP tumour samples (Group A) used in this study, Table S2: The origin of samples of non-tumoured skin from turtles with FP tumours (Group B samples) used in this study, Table S3: The origin of samples of non-tumoured skin from turtles without FP tumours (Group C samples) used in this study. Table S4: The average copy number of ChHV5 in various units calculated in different studies in comparison to the current study.
