# Supplementary material for: The Concurrent Detection of Chelonid Alphaherpesvirus 5 and Chelonia mydas Papillomavirus 1 in Tumoured and Non-Tumoured Green Turtles
Source: Animals (Basel). 2021 Mar 5;11(3):697. doi: 10.3390/ani11030697 (PMC7999010; doi:10.3390/ani11030697)
Supplement: Supplementary file 1 [file animals-11-00697-s001.pdf]

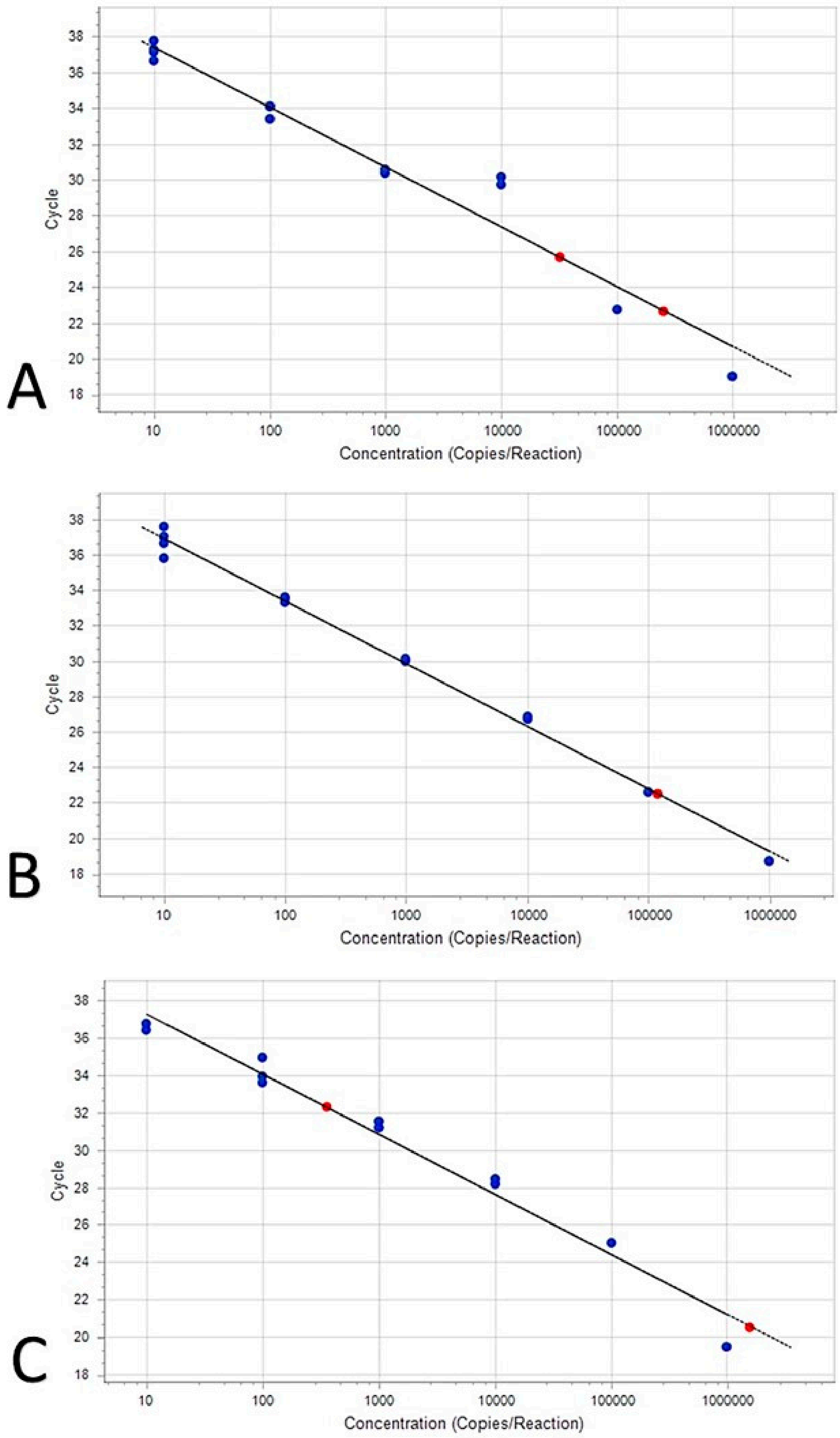

Figure S1. Absolute quantification and the standard curves for three cloned plasmids: A) GAPDH, B) ChHV5 Dpol and C) CmPV1-E1 were plotted based on the cycles (Cq values) and the log of concentration (10 to 10^8 copies per reaction).

Table S1 The origin of FP tumour samples (Group A) used in this study, including location, turtle tag number, curved carapace length (CCL) and weight. All samples were collected from green turtles, excluding three samples from a green/hawksbill hybrid (*) and one sample from a loggerhead (**). The calculated concentration of each target is provided for each sample (where applicable). The copy number per cell of Chelonia mydas papillomavirus 1 (CmPV1) and/or Chelonid alphaherpesvirus 5 (ChHV5) is per sample (shown as appropriate).

		Demographic information (if available)			Calculated Concentration: copy number per reaction (2 μL)			Copy number per cell	
**Sample region**	**Sample ID**	**Tag number**	**CCL (cm)**	**Weight (kg)**	**CmPV1**	**ChHV5**	**GAPDH**	**CmPV1**	**ChHV5**
Townsville	CB83FP	QA29610	45.0	8.0		1.40E+06	1.10E+05		2.55E+01
Townsville	CB93FP	QA36631	59.6	29.0		878573.1	132187.1		13.29287
Townsville	CB94FP	QA9554	53.7	13.1		736462.9	56987.51		25.84647
Townsville	CB95FP	QA29605	57.5	22.0		102.1898	34.12851		5.98853
Townsville	CB96FP	QA32170	61.1	26.0			11295.94		
Townsville	CB97FP	K92985	46.2	-		296245.1	39526.74		14.98961
Townsville	CB98FP	QA15682	49.7	10.6		20.40152	23485.09		0.001737
Townsville	CB99FP1	QA42017	48.6	13.1		540504.7	171407.8		6.306654
Townsville	CB99FP2				54.67283	417018.6	108785.5	0.001005	7.666805
Townsville	CB100FP?	QA38829	42.0	8.2		21.44204	677.4224		0.063305
Townsville	CB101FP1	QA7433	44.9	8.8	384.6	6.52E+05	1.36E+05	5.66E-03	9.59E+00
Townsville	CB101FP2				871.7	3.19E+06	1.95E+05	0.008945	32.69369
Townsville	CB101FP3				37.96307	921489.6	220937.9	0.000344	8.341616
Townsville	CB102FP1	QA47530	48.1	12.7	702.1854	5360.511	67088.33	0.020933	0.159805
Townsville	CB102FP2				133.3026	6554.884	63568.35	0.004194	0.206231
Townsville	CB102FP3				5045	1125	6.47E+04	0.155999	0.034787
Townsville	CB103FP1	QA7392	50.2	12.9	88.65	2.50E+05	2.58E+05	0.000689	1.937864
Townsville	CB103FP2				214.8988	434833	118051.5	0.003641	7.366834
Townsville	CB103FP3				163.0798	234421.1	44396.28	0.007347	10.5604
Townsville	CB104FP1	QA42248	44.2	8.8		808530.2	151929.6		10.64349
Townsville	CB104FP2					134093.7	10722.72		25.01113
Townsville	CB104FP3				52.90798	245960	53662.31	0.001972	9.166954

Townsville	CB105FP1	QA38803	50.5	13.4	74.3866	489490.7	147616.6	0.001008	6.631921
Townsville	CB105FP2				19.25	4.70E+05	2.96E+05	0.00013	3.175397
Townsville	CB106FP1	QA7381	53.0	14.9	80.9203	186848.6	36285.02	0.00446	10.29894
Townsville	CB106FP2				328.3374	298552.9	30872.87	0.02127	19.34079
Townsville	CB106FP3				35.47351	119518.3	55331.87	0.001282	4.320054
Townsville	CB107FP1	QA7388	49.5	13.1		470056.9	104041.2		9.035978
Townsville	CB107FP2					780594	1140585		1.368761
Townsville	CB107FP3				20.8703	252964.3	682013.6	6.12E-05	0.741816
Townsville	CB108FP1	QA42923	43.6	6.5		198.266	9170.036		0.043242
Townsville	CB108FP2				17.05147	214.5014	26865.94	0.001269	0.015968
Townsville	CB108FP3					23.1668	1384.618		0.033463
Townsville	CB109FP1	QA38835	50.3	15.3	694.8	1.96E+05	1.55E+06	0.000897	0.252421
Townsville	CB109FP2				477.2608	95185.5	59923.15	0.015929	3.176919
Townsville	CB109FP3				367.427	81824.71	46010.18	0.015972	3.556809
Townsville	CB110FP1	QA38827	44.0	9.8		491515.1	176833.8		5.559063
Townsville	CB110FP2				27.00222	116724.7	49669.29	0.001087	4.700076
Townsville	CB110FP3				205.3986	795979.2	72820.47	0.005641	21.86141
Townsville	CB111FP1	QA36842	46.3	12.5		522285.4	97321.2		10.73323
Townsville	CB111FP2					526739	97293.38		10.82785
Townsville	CB111FP3					626337.6	136207.9		9.196785
Townsville	CB112FP1	QA47488*	60.3	21.8		3219.141	61.73183		104.2943
Townsville	CB112FP2					23636.45	1442.775		32.76527
Townsville	CB112FP3					63443.51	3841.37		33.03171
Townsville	CB113FP1	No Tag				239.0367	46.51105		10.2787
Townsville	CB113FP2					141.8268	85.7317		3.30862
Townsville	CB113FP3					1974.158	270.6072		14.59058
Townsville	CB114FP1	QA62135	48.0	9.7	59.85474	1338103	152347	0.000786	17.56652
Townsville	CB114FP2				88.32	2.13E+06	2.63E+05	0.000672	16.23431
Townsville	CB114FP3				227.478	478559.2	132242.7	0.00344	7.237587
Townsville	CB115FP	QA9588				836.8713	27311.15		0.061284

Bowen	BW99FP	K59365	46.5	11.6	206.0745	141624.4	62956.17	0.006547	4.499141
Bowen	BW01FP	K52477	48.4	12.0	523.4	8.714	7.88E+04	0.013286	0.000221
Bowen	BW107FP	K97336	25.7	10.5	36.6676	2008167	332241.6	0.000221	12.08859
Bowen	BW108FP	QA15758	49.4	13.0	10.60037	3566.926	17655.55	0.001201	0.404057
Bowen	BW116FP	QA15730	71.6	34.2	29.57064		1517.905	0.038962	
Bowen	BW117FP	QA15763	58.0	21.9			323.4765		
Bowen	BW118FP	QA15774	48.7	11.0	535.6	393.3	1.55E+06	0.000691	0.000507
Bowen	BW119FP	QA15788	53.3	15.6	222		1.85E+04	0.024	
Bowen	BW120FP	QA32132**	85.5	72.4		32681.3	664.4833		98.36606
Bowen	BW121FP	QA36607	73.1	43.0			26796.37		
Bowen	BW122FP	QA36634	57.4	22.5	177.6	15.31	8.49E+04	0.004186	0.000361
Bowen	BW123FP	QA7340	45.3	10.2		381673.2	38733.7		19.70755
Bowen	BW124FP1	QA36626	44.8	-	47.64	5.57E+04	3.01E+05	0.000317	0.370259
Bowen	BW124FP2				124.4032	9514.502	38719.79	0.006426	0.491454
Bowen	BW125FP?	QA36635	55.9	18.0	91.28411		1160.759	0.157284	
Bowen	BW126FP1	QA36636	47.6	11.8	139	540	6.37E+04	0.004365	0.016957
Bowen	BW126FP2					504.0661	32806.78		0.030729
Bowen	BW127FP	K79524	95.1	93.3		144.0536	465.1105		0.619438
Bowen	BW128FP	K93052	42.5	8.5	282.977	13146.21	56709.25	0.00998	0.463635
Bowen	BW129FP	K94004	62.1	28.0	58.69069		1059.89	0.110749	
Bowen	BW130FP	K97113	47.9	-	34.98911	1500457	441319.3	0.000159	6.799871
Bowen	BW131FP	K97114	50.5	-		1001654	134246.2		14.92265
Bowen	BW132FP	K97117	45.0	-		1266036	66100.51		38.30639
Bowen	BW134FP	K97287	57.0	-		1161.984	423.0933		5.492801
Bowen	BW135FP	K97288	46.2	-		19154.51	823.3694		46.52715
Bowen	BW136FP	K97289	51.8	-	31.38807	977766.9	114030.7	0.000551	17.14919
Bowen	BW137FP	QA15638	48.6	11.0		711360.8	53662.31		26.51249
Bowen	BW138FP	QA15672	45.3	10.6	92.18531		218.2944	0.844596	
Bowen	BW139FP	QA15678	76.2	43.6	81.8215	526334.1	116521.1	0.001404	9.034143
Bowen	BW140FP	QA15761	77.1	54.6	18.1		1.73E+05	0.000209	

Bowen	BW141FP	QA15787	43.1	9.2			735.9959		
Bowen	BW142FP	QA15951	45.4	-		1015825	112528.1		18.0546
Bowen	BW143FP	QA24175	45.5	9.7	48.73993	10522.63	23596.39	0.004131	0.891885
Bowen	BW144FP	QA29702	44.4	10.0		1783868	37523.27		95.08061
Bowen	BW145FP	QA36630	47.8	13.0		959.5476	73.93351		25.95704
Bowen	BW146FP?	QA47548	42.1	9.4		5716.798	116980.2		0.09774
Bowen	BW147FP?	K52483	52.3	14.8			40.52847		
Bowen	BW148FP?	K97048	46.6	-			118.6637		
Bowen	BW149FP?	K97070	69.8	35.7			218.5727		
Bowen	BW150FP?	QA15666	46.9	12.0		108.8702	265.1811		0.821101
Bowen	BW151FP	QA9536	44.6	8.4			38.6224		
Bowen	BW151FP?						466.9192		
Bowen	BW152FP	K52464	54.0	-	989.7	6731	4.58E+05	0.004327	0.029425
Bowen	BW153FP	K92663	44.7	-	56.70054	994366.6	649735.5	0.000175	3.060835
Bowen	BW154FP	K93074	45.0	11.0		1050644	2893897		0.72611
Bowen	BW155FP	K93640	47.9	11.8		3464088	1747469		3.964693
Bowen	BW156FP	K97025	48.1	-	2.74E+05	229.2	5.20E+05	1.052915	0.000882
Bowen	BW158FP	K97483	48.5	-		4198527	357145.9		23.51155
Bowen	BW159FP	QA15979	47.2	-	691.5	6.75E+07	1.07E+06	0.001289	125.8155
Bowen	BW160FP	QA15980	44.0	-	700.6	5.46E+06	6.30E+05	0.002224	17.33333
Bowen	BW162FP	K93038			289.6609	10591.46	396380.4	0.001462	0.053441
Bowen	9231	09-231	57.1	-		7890963	1897729		8.316219
Bowen	QA4962		44.5	-		3593647	440901.9		16.30135
Brisbane	MB01FP1	No tag 1	46.3	-		3357202	1426079		4.708297
Brisbane	MB01FP2			-	138.2	2.96E+06	2.63E+05	0.001052	22.53521
Brisbane	MB01FP3			-	76.03	3.60E+06	2.39E+05	0.000636	30.07525
Brisbane	MB03FP1	No tag 3	44.4			1341747	380937		7.044456
Brisbane	MB03FP2					1085463	428380.2		5.067754
Brisbane	MB03FP3					1793180	168068.6		21.33866
Brisbane	MB04FP1	No tag 4	43.1	-		1473331	644726.9		4.570403

Brisbane	MB04FP2					1671718	366606.7		9.119955
Brisbane	MB05FP	QA45711	52.1	-		526739	130350.6		8.081882
Brisbane	MB06FP1	K87178	62.3	-			1102.324		
Brisbane	MB07FP1	Kerry			30.86612	2760.825	24.33378	2.536895	226.913
Brisbane	SW01FP1	Alice	45.0	-		1464828	1452514		2.016956
Brisbane	SW01FP2					1200042	555127.4		4.323483
Brisbane	SW02FP1	Tay	52.4	-		421877	68090.06		12.39174
Brisbane	SW02FP2				69.20469	3409431	133954	0.001033	50.90448
Gladstone	GS52FP1	QA34793	101.1	126.0	465.8	873.4	1.73E+05	0.005388	0.010103
Gladstone	GS72FP	QA58207	60.2	24.0	30.16769	124822.2	60702.27	0.000994	4.112603
Gladstone	GS73FP	QA58252	60.3	23.3			63999.65		
Gladstone	GS74FP	QA58271	70.1	36.5			87067.35		
Cairns	CN01FP1	Roxy	47.6	-	280.9117	15494467	295511.4	0.001901	104.8654
Cairns	CN01FP2				52.11943	4125650	1471992	7.08E-05	5.605533
Cairns	CN02FP1	Destiny			150.3	1.99E+05	5.35E+04	0.005621	7.430815
Airlie Beach	AB01FP1	No tag	49.0	-		615406.1	360485		3.414323
Airlie Beach	AB01FP2				163.9059	419042.9	345041.6	0.00095	2.428942
Airlie Beach	AB01FP3				57.86459	822700.7	397632.6	0.000291	4.137994
Airlie Beach	AB01FP4					868046.4	409597.7		4.238531

Table S2 The origin of samples of non-tumoured skin from turtles with FP tumours (Group B samples) used in this study, including location, turtle tag number, curved carapace length (CCL) and weight. All samples were collected from green turtles, excluding one sample from a loggerhead (Caretta caretta) (**). The calculated concentration of each target is provided for each sample (where applicable). The copy number per cell of Chelonia mydas papillomavirus 1 (CmPV1) and/or Chelonid alphaherpesvirus 5 (ChHV5) is per sample (shown as appropriate).

		Demographic information (if available)			Calculated Concentration: copy number per reaction (2 μL)			Copy number per cell	
**Sample region**	**Sample ID**	**Tag number**	**CCL (cm)**	**Weight (kg)**	**CmPV1**	**ChHV5**	**GAPDH**	**CmPV1**	**ChHV5**
Townsville	CB83N	QA29610	45.0	8.0			152.7935		
Townsville	CB99N	QA42017	48.6	13.1			4984.502		
Townsville	CB101N	QA7433	44.9	8.8			251.6029		
Townsville	CB102N	QA47530	48.1	12.7			9585.92		
Townsville	CB103N	QA7392	50.2	12.9			2382.61		
Townsville	CB104N	QA42248	44.2	8.8			6637		
Townsville	CB105N	QA38803	50.5	13.4			381.9397		
Townsville	CB106N	QA7381	53.0	14.9		14.03	2.06E+04		0.001361
Townsville	CB107N	QA7388	49.5	13.1			152.7935		
Townsville	CB108N	QA42923	43.6	6.5	247		1.03E+04	0.047776	
Townsville	CB109N	QA38835	50.3	15.3			689.5522		
Townsville	CB110N	QA38827	44.0	9.8			168.9975		
Townsville	CB111N	QA36842	46.3	12.5	11.29	7756	2.01E+05	1.13E-04	0.077289
Bowen	BW01N	K52477	48.4	12.0			1.59E+04		
Bowen	BW99N	K59365	46.5	11.6	5545		4.36E+04	0.254241	
Bowen	BW107N	K97336	25.7	10.5			1894.727		
Bowen	BW108N	QA15758	49.4	13.0		113	5107.794	0.000163	0.002029
Bowen	BW116N	QA15730	71.6	34.2			4025.03		
Bowen	BW117N	QA15763	58.0	21.9			5270.715		
Bowen	BW118N	QA15774	48.7	11.0			2045.76		
Bowen	BW119N	QA15788	53.3	15.6	9.80E+04		2.60E+04	7.535563	
Bowen	BW120N	QA32132**	85.5	72.4	29.30379		575.9478	0.101758	

Bowen	BW121N	QA36607	73.1	43.0			8326.585		
Bowen	BW122N	QA36634	57.4	22.5	6140		2.73E+04	0.450312	
Bowen	BW124N	QA36626	44.8	-			5151.827		
Bowen	BW125N	QA36635	55.9	18.0	46.79859		4460.513	0.020983	
Bowen	BW126N	QA36636	47.6	11.8	143.9676		4905.244	0.058699	
Bowen	BW145N	QA36630	47.8	13.0			559.6557		
Gladstone	GS52N	QA34793	101.1	126.0	5089		1.15E+05	0.088427	
Gladstone	GS72N	QA58207	60.2	24.0			3375.547		
Gladstone	GS73N	QA58252	60.3	23.3			2107.846		
Gladstone	GS74N	QA58271	70.1	36.5			2542.889		
Brisbane	MB01N	No tag 1	46.3	-		25.52748	47467.26		0.001076
Brisbane	MB03N	No tag 3	44.4	-			1.04E+04		
Brisbane	MB05N	QA45711	52.1	-			2341.659		
Brisbane	MB06N	K87178	62.3	-			638.0339		

Table S3 The origin of samples of non-tumoured skin from turtles without FP tumours (Group C samples) used in this study, including location, turtle tag number, curved carapace length (CCL) and weight. All samples were collected from green turtles. The calculated concentration of each target is provided for each sample (where applicable). The copy number per cell of Chelonia mydas papillomavirus 1 (CmPV1) and/or Chelonid alphaherpesvirus 5 (ChHV5) is per sample (shown as appropriate).

		Demographic information (if available)			Calculated Concentration: copy number per reaction (2 μL)			Copy number per cell	
**Sample region**	**Sample ID**	**Tag number**	**CCL (cm)**	**Weight (kg)**	**CmPV1**	**ChHV5**	**GAPDH**	**CmPV1**	**ChHV5**
Townsville	CB01	QA32220	44.7	9.6			7.47E+10		
Townsville	CB02	K92947/QA29615	47.4	10.0			1054004		
Townsville	CB03	QA32265	48.3	48.0	472.9		1.75E+11	5.42E-09	
Townsville	CB04	QA29616	66.4	30.0			4.71E+10		
Townsville	CB05	K56931	46.6	12.0			2.03E+11		
Townsville	CB06	K18987	46.3	11.0			9.76E+09		
Townsville	CB07	QA29706	42.7	8.2			115230.7		
Townsville	CB09	QA32222	54.3	17.1	135.2		415974.6	0.00065	
Townsville	CB10	QA29603	48.4	13.0			1750133		
Townsville	CB11	QA29709	62.5	-			1038195		
Townsville	CB12	QA9469	45.2	9.5			210205		
Townsville	CB13	QA29712	75.7	-			147270.7		
Townsville	CB14	QA29711	45.4	10.8			187592.2		
Townsville	CB16	QA29604	52.2	16.5			261816.5		
Townsville	CB18	QA29707	48.0	13.6			599831.6		
Townsville	CB20	QA32206	46.9	10.5	135.8		782863.6	0.000347	
Townsville	CB21	QA29601	48.6	13.0			271393.4		
Townsville	CB22	K90948	43.2	9.0	7452		182648.2	0.0816	
Townsville	CB23	QA32212	42.2	8.8	546.1		202033.1	0.005406	
Townsville	CB24	QA32215	69.4	-			291322.8		
Townsville	CB25	QA32213	46.5	10.8			1.75E+11		
Townsville	CB26	QA9595	50.9	15.0			4.71E+10		

Townsville	CB27	QA29605	57.4	22.0			2.03E+11		
Townsville	CB28	K92803	83.2	-			9.76E+09		
Townsville	CB29	K92644	60.0	-			1.09E+10		
Townsville	CB30	K92999	47.2	-	240.7		484542.3	0.000994	
Townsville	CB31	QA32211/K40368	44.2	8.5	107		759056.1	0.000282	
Townsville	CB32	K59356	45.2	10.2	653.4		1581766	0.000826	
Townsville	CB33	QA29620	48.7	14.5			87366.36		
Townsville	CB34	K92670	70.8	-			317240.5		
Townsville	CB35	QA32242	85.8	-		88.7	1046292		0.00017
Townsville	CB36	QA32275	73.6	-		2.22E+05	116871.9		3.792186
Townsville	CB37	QA32250	86.6	-		55.47	125619.5		0.000883
Townsville	CB38	QA32234	71.8	-			698437.2		
Townsville	CB39	K92985	46.2	-			144322.9		
Townsville	CB40	QA15846	103.9	-			115230.7		
Townsville	CB41	K90921	40.8	7.1			69273.49		
Townsville	CB42	QA15682	49.7	10.6			274325.9		
Townsville	CB43	K92980	46.4	9.6			176742.8		
Townsville	CB44	QA32300	48.5	11.8			162074.6		
Townsville	CB45	K92981	50.7	11.9	87.23		254332.9	0.000686	
Townsville	CB46	K92982	45.2	9.1			374370.4		
Townsville	CB47	K92990	74.6	-	1189		283515.5	0.008388	
Townsville	CB48	QA9280	100.9	-			1.52E+05		
Townsville	CB49	QA36779	46.6	-			1.1E+09		
Townsville	CB50	QA9279	48.8	-			1.09E+10		
Townsville	CB51	QA9528	43.9	-			4.39E+05		
Townsville	CB52	QA9283	46.3	-			3.93E+05		
Townsville	CB53	K88974	68.5	-			6.11E+05		
Townsville	CB54	K92949	48.9	12.1			115230.7		
Townsville	CB55	QA9277	47.8	-			5.97E+10		

Townsville	CB56	K74948	55.7	-		131.6	9.95E+05		0.000264
Townsville	CB57	QA36799	50.4	-		1.42E+05	1.44E+11		1.97E-06
Townsville	CB58	QA36776	93.2	-			3.81E+10		
Townsville	CB59	QA36778	108.1	-			1.46E+11		
Townsville	CB60	K92989	68.7	-	106.6		6.84E+09	3.12E-08	
Bowen	BW02	QA15707	46.8	10.8			3.23E+05		
Bowen	BW03	QA29729	45.9	11.4			1.18E+06		
Bowen	BW04	QA36605	113.9	130+			7.21E+05		
Bowen	BW05	QA15776	45.0	8.7	1424		1.69E+05	0.016902	
Bowen	BW06	QA15785	49.1	10.5			1.07E+05		
Bowen	BW07	K59353	60.6	21.9	12.63		1.45E+05	0.000175	
Bowen	BW08	K59391	45.5	9.5	93.03		1.58E+05	0.001181	
Bowen	BW09	K92959	42.5	7.7			1.90E+05		
Bowen	BW10	QA15796	54.0	17.5	25.13		3.04E+05	0.000165	
Bowen	BW11	QA36603	80.5	48.0			4.39E+05		
Bowen	BW12	QA36606	85.7	61.0		164.4	3.93E+05		0.000838
Bowen	BW13	QA15777	44.4	7.9			6.11E+05		
Bowen	BW14	QA32125	42.3	9.1			2.13E+05		
Bowen	BW15	QA32123	48.1	12.6			1.58E+05		
Bowen	BW16	QA15968	47.0	11.6			1.64E+05		
Bowen	BW17	K52467	58.5	22.2	1.36E+04		2.38E+05	0.114262	
Bowen	BW18	K94004	63.0	25.1	6286		8.93E+04	0.140863	
Bowen	BW19	K92945	46.0	10.2	67.85		4.44E+04	0.003056	
Bowen	BW20	QA15794	49.0	12.1	11.21		5.08E+04	0.000442	
Bowen	BW21	QA15782	58.0	19.3	105.2		1.82E+05	0.001154	
Bowen	BW22	QA15799	64.0	23.6	100.2		2.54E+05	0.00079	
Bowen	BW23	K97074	97.0	97.1		4129	3.86E+05		0.021394
Bowen	BW24	QA15769	97.0	100.2	12.95		5.81E+05	4.46E-05	
Bowen	BW25	K97161	89.0	74.8		29.07	1.29E+06		4.50E-05
Bowen	BW26	QA15732	82.3	60.8		46.72	7.40E+04		0.001263

Bowen	BW27	QA10912	103.3	126.5	21.16		3.20E+05	0.000132	
Bowen	BW28	QA15748	97.5	94.0	504.9		1.00E+06	0.001006	
Bowen	BW29	QA15746	106.5	134.3			1.21E+05		
Bowen	BW30	K97284	53.8	13.3	13.29	8401	1.43E+05	0.000185	0.117251
Bowen	BW31	QA15733	64.5	30.9			6.00E+05		
Bowen	BW32	QA15766	43.7	8.5			1.37E+05		
Bowen	BW33	QA15740	65.9	29.5			1.19E+05		
Bowen	BW34	K59377	44.5	10.1		17.18	7.21E+04		0.000476
Bowen	BW35	QA15717	47.6	10.3			2.60E+05		
Bowen	BW36	QA15709	44.4	8.3	38.34	11.1	1.51E+05	0.000506	0.000147
Bowen	BW37	K59378	47.5	12.2	208.2		1.55E+05	0.00268	
Bowen	BW38	QA15718	49.2	13.7	276.1		2.47E+05	0.00224	
Bowen	BW39	QA15770	44.5	8.2	54.92		3.59E+05	0.000306	
Bowen	BW40	QA15775	43.5	10.0			2.92E+05		
Bowen	BW41	QA15747	45.1	10.0	18.55		1.52E+05	0.000244	
Bowen	BW42	QA15710	56.5	16.9			1.02E+09		
Bowen	BW43	QA15764	47.5	12.5	27.08		7.30E+08	7.42E-08	
Bowen	BW44	QA15767	49.3	13.9			6.14E+08		
Bowen	BW45	K97135	46.8	12.5	129.3	1143	1.11E+08	2.34E-06	2.07E-05
Bowen	BW46	QA15731	55.0	19.9	3.66E+04		2.26E+08	0.000324	
Bowen	BW47	QA15736	41.2	7.4	46.8		1.15E+07	8.13E-06	
Bowen	BW48	K59376	57.5	18.9	696.7		9.07E+08	1.54E-06	
Bowen	BW49	QA15771	47.4	11.2	108.4		1.85E+09	1.18E-07	
Bowen	BW50	K59383	45.8	9.9	75.02		1.04E+08	1.44E-06	
Bowen	BW51	K59381	48.4	13.0	111.8		3.82E+07	5.85E-06	
Bowen	BW52	QA32101	44.7	10.5	275.4		8.65E+07	6.37E-06	
Bowen	BW53	QA29752	42.7	9.8	1043		1.74E+08	1.20E-05	
Bowen	BW54	K52467	57.4	21.8	194.3		3.27E+08	1.19E-06	
Bowen	BW55	QA15783	44.8	11.8	186.7		5.59E+08	6.69E-07	
Bowen	BW56	K52461	61.5	28.0			1.64E+09		

Bowen	BW57	K59382	58.0	22.0	973.7		4.89E+08	3.98E-06	
Bowen	BW58	QA36658	44.1	10.0	112.1		2.58E+09	8.70E-08	
Bowen	BW59	QA36604	46.8	11.0			1.66E+08		
Bowen	BW60	QA36612	93.5	86.0	35.99		4.64E+07	1.55E-06	
Bowen	BW61	QA36655	45.5	12.0			3.55E+08		
Bowen	BW62	QA36820	46.7	13.0	61.73		1.27E+09	9.75E-08	
Bowen	BW63	QA32124	57.0	22.5	539.8	952.4	7.63E+07	1.42E-05	2.50E-05
Bowen	BW64	QA24108	48.6	12.6	1.02E+04		4.74E+08	4.31E-05	
Bowen	BW65	QA29731	65.6	34.5	220.7	11.16	4.91E+08	9.00E-07	4.55E-08
Bowen	BW66	QA15716	67.8	34.0	29.72		2.00E+08	2.97E-07	
Bowen	BW67	QA36625	55.1	23.0	1318		5.57E+08	4.73E-06	
Bowen	BW68	QA32120	56.5	20.4	1848		6.34E+08	5.83E-06	
Bowen	BW69	QA29745	50.6	14.5	3.36E+05		6.83E+08	0.000983	
Bowen	BW70	QA36611	51.6	14.5			4.33E+08		
Bowen	BW71	QA32122	37.2	13.1	91.59		1.86E+07	9.83E-06	
Bowen	BW72	QA36608	43.7	12.5	467.6		4.80E+08	1.95E-06	
Bowen	BW73	QA36656	43.5	10.2	86.26		3.95E+08	4.37E-07	
Bowen	BW74	QA29730	44.2	10.7	933.8		6.39E+08	2.92E-06	
Bowen	BW75	QA36676	44.3	9.5	52.07		1.28E+08	8.16E-07	
Bowen	BW76	QA36679	45.8	9.0	96.83		3.15E+08	6.15E-07	
Bowen	BW77	QA36677	43.0	10.0	33.6	484.6	4.24E+07	1.59E-06	2.29E-05
Bowen	BW78	QA36684	51.4	16.0	253.5		7.92E+07	6.40E-06	
Bowen	BW79	QA15641	53.6	17.6	2009		6.74E+08	5.96E-06	
Bowen	BW80	QA32109	43.7	20.6		1.97E+04	1.10E+09		3.60E-05
Bowen	BW81	QA32104	47.5	21.6	28.97		3.33E+08	1.74E-07	
Gladstone	GS01	QA33350	42.1	7.5			2.51E+10		
Gladstone	GS02	QA33349	42.5	8.9		3.64E+04	4.13E+12		1.76E-08
Gladstone	GS03	QA33342	111.0	153.0		2217	7.47E+11		5.93E-09
Gladstone	GS04	QA33363	107.4	141.0		1579	7.07E+12		4.47E-10
Gladstone	GS05	QA33340	96.3	97.0		36.32	1.56E+12		4.66E-11

Gladstone	GS06	QA33373	99.4	117.0			2.13E+12		
Gladstone	GS07	QA33352	88.3	94.0		601.9	6.42E+10		1.88E-08
Gladstone	GS08	QA33341	95.1	88.0		1186	4.72E+11		5.03E-09
Gladstone	GS09	QA33332	79.6	55.0		327.7	2.47E+12		2.66E-10
Gladstone	GS10	QA33330	98.0	108.0		666.4	2.52E+12		5.29E-10
Gladstone	GS11	QA33357	106.0	128.0		6170	1.28E+12		9.66E-09
Gladstone	GS12	QA33343	95.4	97.0		2.19E+05	1.82E+12		2.40E-07
Gladstone	GS13	QA39981	100.3	103.0		5.44E+04	2.12E+12		5.12E-08
Gladstone	GS14	QA33354	87.1	66.0		183.2	3.24E+12		1.13E-10
Gladstone	GS15	QA33333	79.7	53.5		1845	4.44E+12		8.31E-10
Gladstone	GS16	QA33329	63.9	30.0			5.01E+12		
Gladstone	GS17	QA34529	47.9	11.8			3.38E+14		
Gladstone	GS18	QA33337	48.5	14.1			2.70E+10		
Gladstone	GS19	QA33370	47.5	11.4		536.4	1.21E+07		8.87E-05
Gladstone	GS20	QA33365	60.0	22.9			2.38E+11		
Gladstone	GS21	QA33368	46.0	11.8			9.38E+07		
Gladstone	GS22	QA33367	52.1	17.0			5.77E+12		
Gladstone	GS23	QA33375	49.1	14.7		216.3	3.52E+08		1.23E-06
Gladstone	GS24	K70229	105.7	142.0		110.9	4.51E+12		4.92E-11
Gladstone	GS25	QA43067	105.7	140.0			176742.8		
Gladstone	GS26	QA43063	93.5	93.0			162074.6		
Gladstone	GS27	QA43068	96.1	106.0			254332.9		
Gladstone	GS28	QA43065	97.7	96.0			2.52E+12		
Gladstone	GS29	QA43056	52.7	17.8			1.28E+12		
Gladstone	GS30	QA43061	84.3	75.0			1.82E+12		
Gladstone	GS31	QA43060	74.8	45.0			5.56E+13		
Gladstone	GS32	QA43055	85.1	75.0			1.54E+12		
Gladstone	GS33	QA43058	88.7	72.0		14.38	1.15E+12		2.49E-11
Gladstone	GS34	QA43059	99.5	120.0			7.55E+11		
Gladstone	GS35	QA27998	41.6	7.6	385.5		1.31E+10	5.89E-08	

Gladstone	GS36	QA43053	44.5	8.6	6688		1.90E+11	7.05E-08	
Gladstone	GS37	QA27997	60.0	19.0	141.9		4.15E+10	6.84E-09	
Gladstone	GS38	QA43044	43.1	6.5			5.37E+09		
Gladstone	GS39	QA43043	42.6	6.2			4.01E+10		
Gladstone	GS40	QA43023	102.7	131.0			1.04E+12		
Gladstone	GS41	QA43031	59.8	26.0			7.64E+09		
Gladstone	GS42	QA43022	90.6	82.0		18.86	1.09E+10		3.45E-09
Gladstone	GS43	QA43033	56.0	21.0		169.2	2.59E+09		1.31E-07
Gladstone	GS44	QA43036	80.6	64.0	66.39	8356	2.12E+09	6.27E-08	7.89E-06
Gladstone	GS45	QA43041	94.3	92.0		850.8	3.37E+09		5.05E-07
Gladstone	GS46	QA43003	43.3	8.8		86.45	5.58E+07		3.10E-06
Gladstone	GS47	QA43004	93.8	104.0			7.38E+09		
Gladstone	GS48	QA43035	64.6	32.0			9.29E+09		
Gladstone	GS49	QA34580	46.2	10.8	62.42		6.22E+06	2.01E-05	
Gladstone	GS50	QA34576	43.4	9.9	593.1		1.09E+10	2.09E-03	

Table S4 The average copy number of ChHV5 in various units calculated in different studies in comparison to the current study. The copy number per reaction of GAPDH is consistent in samples from green turtles with and without FP tumours. In other words, there is no significant difference between the quantities of tissues used for the qPCR reactions and subsequently viral load comparison (P value: 0.282). As the starting tissue mass appeared to be similar in samples, after calculating the DNA extraction elute volume (50μL of extracted DNA in DNase free water to 20μL of PCR reaction) the estimated mean of ChHV5 viral loads appears to be approximately 3.707×105 copies/μL of DNA extract from tumour tissues. This unit calculation enabled us to compare our results of ChHV5 viral loads with previous studies that used different protocols and calculated copies/cell, copies/μL of samples in PCR reaction or copies/μgr of DNA (Table S7). Overall, the results in this study are consistent with studies in other regions.

			Quackenbush *et al.*, 2001	Greenblatt, *et al.*, 2005	Page-Karjian *et al.*, 2015	Alfaro-Núñez *et al.*, 2016	Current study
FP-afflicted turtles	FP Tumour Samples (Group A in the present study)	Copies/cell	15.21	13.9	-	15.61 ± 12.5	15.635± 30.152
		copies/μL of samples in PCR reaction	-	-	-	4.9×10^{4}	4.297×10^{5}
		copies/μg of DNA	3.3×10^{4} to 4.9×10^{6}	-	3.24×10^{8} (1.6×10^{3} -2.0×10^{9})	-	5.367×10^{5}
	Non-tumoured skin from turtles with FP tumours (Group B in the present study)	Copies/cell	-	0.03[$]	-	0.02 ± 0.00	0.020± 0.0379
		copies/μL of samples in PCR reaction	-	-	-	1.8×10^{1}	1.818×10^{2}
		copies/μg of DNA	122 ± 82.67	-	22722 (2.5×10^{2} - 7.1×10^{4})	-	2.272×10^{3}
Non-tumoured turtles (Group C in the present study)		Copies/cell	-	-	-	6.03	0.100± 0.606
		copies/μL of samples in PCR reaction	-	-	-	5.8×10^{4}	2.614×10^{3}
		copies/μg of DNA	-	-	5463 (6.0×10^{2} - 1.7×10^{4})	-	3.269×10^{3}
